# Theta and Alpha Oscillatory Activity During Working Memory Maintenance in Long-Term Cannabis Users: The Importance of the Polydrug Use Context

**DOI:** 10.3389/fnhum.2021.740277

**Published:** 2021-10-18

**Authors:** Alicja Anna Binkowska, Natalia Jakubowska, Klaudia Krystecka, Natalia Galant, Agnieszka Piotrowska-Cyplik, Aneta Brzezicka

**Affiliations:** ^1^SWPS University of Social Sciences and Humanities, Warsaw, Poland; ^2^Polish-Japanese Academy of Information Technology, Warsaw, Poland; ^3^Nencki Institute of Experimental Biology PAS, Warsaw, Poland; ^4^ Institute of Forensic Genetics, Bydgoszcz, Poland; ^5^ Institute of Food Technology of Plant Origin, Poznań University of Life Sciences, Poznań, Poland

**Keywords:** EEG, cannabis, alpha, theta, WM, polydrug use, oscillations

## Abstract

**Background:** Impairments in various subdomains of memory have been associated with chronic cannabis use, but less is known about their neural underpinnings, especially in the domain of the brain’s oscillatory activity.

**Aims:** To investigate neural oscillatory activity supporting working memory (WM) in regular cannabis users and non-using controls. We focused our analyses on frontal midline theta and posterior alpha asymmetry as oscillatory fingerprints for the WM’s maintenance process.

**Methods:** 30 non-using controls (CG) and 57 regular cannabis users—27 exclusive cannabis users (CU) and 30 polydrug cannabis users (PU) completed a Sternberg modified WM task with a concurrent electroencephalography recording. Theta, alpha and beta frequency bands were examined during WM maintenance.

**Results:** When compared to non-using controls, the PU group displayed increased frontal midline theta (FMT) power during WM maintenance, which was positively correlated with RT. The posterior alpha asymmetry during the maintenance phase, on the other hand, was negatively correlated with RT in the CU group. WM performance did not differ between groups.

**Conclusions:** Both groups of cannabis users (CU and PU), when compared to the control group, displayed differences in oscillatory activity during WM maintenance, unique for each group (in CU posterior alpha and in PU FMT correlated with performance). We interpret those differences as a reflection of compensatory strategies, as there were no differences between groups in task performance. Understanding the psychophysiological processes in regular cannabis users may provide insight on how chronic use may affect neural networks underlying cognitive processes, however, a polydrug use context (i.e., combining cannabis with other illegal substances) seems to be an important factor.

## Introduction

Working memory (WM) system is a foundation of cognitive processes as it allows us to temporarily store and manipulate information, even when it is no longer present in the sensory environment (Baddeley, 2002). EEG studies of WM have increased our understanding of how different brain oscillations relate to information processing in WM (for review: Pavlov and Kotchoubey, 2020). While some research in this field has focused on the oscillatory analyses during overall performance, the other focused on particular phases such as encoding, maintenance or retrieval. Maintenance process, when the information is actively retained and rehearsed, was of particular interest in our study. Sternberg task (Sternberg, 1966) is a well-known procedure to investigate particular WM processes as it gives researchers the opportunity to isolate the period of maintenance from other phases of WM as the stimulus presentation, retention (maintenance), and test phase are temporally separated.

Frontal midline (FM) theta (4–8 Hz) activity has been related to WM processes as it’s power typically increases linearly with increasing memory load in the Sternberg task (e.g., Jensen and Tesche, 2002; Onton et al., 2005). Studies on animals and humans provided further evidence that prefrontal theta oscillations during WM maintenance could reflect prefrontal-hippocampal communication associated with successful memory encoding, long-term potentiation and learning (Klimesch, 1999; Benchenane et al., 2010), as well as successful maintenance of information in WM (Itthipuripat et al., 2013).

Besides theta, also an alpha oscillatory (8–12 Hz) activity over the posterior areas of the brain was shown to be load-dependent during maintenance of information in WM (for review: Pavlov and Kotchoubey, 2020). Maintenance of information in WM was associated with increased alpha power in posterior areas (Jensen et al., 2002; Hu et al., 2019; Proskovec et al., 2019), but decrease in alpha power was also observed in some studies (Stephane et al., 2010; Kwon et al., 2015). The enhancement of occipital alpha power is thought to reflect active inhibition or suppression of incoming sensory input that would interfere with the currently maintained information, so it is providing a sensory gating mechanism—the higher the memory load, the stronger the need for enhanced sensory gating (Jensen et al., 2002; Klimesch et al., 2007; Sauseng et al., 2009).

Previous studies have shown that acute cannabis administration in humans induced a decrease in EEG theta power both in rest and during a working memory task, and led to impairment in WM performance (Ilan et al., 2004, 2005). Further research has shown dose-dependent effects of THC on resting state EEG theta power and Sternberg WM task performance (Böcker et al., 2010).

One of the most consistent and prominent reported acute effects of cannabis are impairments in working memory. As it was shown in recent meta-analysis reported effect size was medium ∼0.5 (Zhornitsky et al., 2020). Moreover, impairments in working memory have been associated with chronic cannabis use (lasting beyond the intoxication phase) as well (Broyd et al., 2016; Curran et al., 2016; Figueiredo et al., 2020). Such deficits in WM were shown in few meta-analyses concentrated on the potential long-term effects of cannabis use, the estimated effect sizes were smaller than in studies on acute effects of cannabis but significant (Grant et al., 2003; Schreiner and Dunn, 2012; Scott et al., 2018; Figueiredo et al., 2020).

Less is known about the neural underpinnings of WM impairments in chronic cannabis users. Δ9-tetrahydrocannabinol (THC), the main psychoactive compound of cannabis, is a partial agonist of CB1 receptors (Fantegrossi et al., 2014). Changes in CB1 receptor signaling (downregulation) are believed to contribute to the cognitive deficits resulting from chronic exposure to cannabis (Hirvonen et al., 2012; D’Souza et al., 2016). Cognitive deficits observed in chronic users seem to be reversible and do not last beyond 4 weeks after cannabis abstinence, following CB1 density normalization (Schreiner and Dunn, 2012; D’Souza et al., 2016; Scott et al., 2018). There is a high density of cannabinoid receptors in key areas of the brain involved in memory processes, such as the hippocampus and prefrontal cortex (PFC) (Curran et al., 2016).

Results of fMRI studies have shown that cannabis users, in comparison to non-users, show hyperactivation of prefrontal (during maintenance phase; Tervo-Clemmens et al., 2018), cingulate and parietal cortex during working memory tasks, despite normal performance (Kanayama et al., 2004; Jager et al., 2006; Nestor et al., 2008; Smith et al., 2010; Tervo-Clemmens et al., 2018; Sagar and Gruber, 2019; Hatchard et al., 2020). These results are in line with the hypothesis that cannabis users put additional effort in task performing, which is mirrored in higher working memory network activations. To the best of our knowledge, there are no published EEG nor MEG studies investigating the chronic/residual effects of cannabis on working memory functions, especially on the maintenance process. Due to their high temporal resolution, EEG and MEG have been important tools for the investigation of oscillatory dynamics related to the critical WM phases, where improper neural activations may lead to memory failures. Previous studies have shown an altered pattern of resting-state oscillations in abstinent chronic cannabis users—reduction in theta (4–7 Hz), alpha1 (8–10 Hz), alpha 2 (10–13 Hz) and beta 2 (25–50 Hz) power compared with non-users (Herning et al., 2003, 2008; Skosnik et al., 2016). However, a recent study found reduced delta power and increased theta, beta, and gamma power in cannabis users compared to control group. Such a pattern of EEG activity suggests increased cortical activity which could indicate a loss of neural refinement and efficiency, and may alter cognitive functioning (Prashad et al., 2018).

Considering the literature above, there is evidence that the neural circuitry serving working memory may be affected in chronic cannabis users. To investigate the relationship between cannabis use and psychophysiology of working memory, we recruited cannabis users and non-using controls.

Detailed analysis of our collected data on self-reported substance use as well as the hair sample analysis (results were delivered after study accomplishment) revealed that the majority of recruited cannabis users actually use other illicit psychoactive substances as well. This occured to be in line with other research reports, which tells that cannabis is the most commonly used drug within a polydrug context [Mitchell and Plunkett, 2000; Carlson et al., 2005; Lynskey et al., 2006; World Health Organization, 2016]. At the same time, it is quite surprising that studies including a polydrug group in comparisons are hard to find in the field of research on cannabis impact on cognition, so it is possible that these two subgroups are often mixed up and treated as a homogenous cannabis users group. Based on combined self-report and objective substance use measurements we decided to divide cannabis users participating in our study into two groups: cannabis users (CU) and polydrug cannabis users (PU, using cannabis and at least one other illicit substance in the last 3 months). Such an approach should provide higher ecological validity and better understanding of memory processes and its neural correlates in cannabis and polydrug cannabis use.

The aim of our study was to investigate the relationship between working memory and regular cannabis use (including cannabis polydrug use) and to assess the neural oscillatory dynamics during information maintenance in users and the controls. We wanted to check if the working memory task performance is affected in regular cannabis users compared to non-using controls and if the polydrug context is an important factor? We have also asked a question whether there are differences in oscillatory brain activity during information maintenance between these groups of users and the control group? We focused our analyses on the frontal midline (FM) theta and posterior alpha oscillatory activity as those two bands show the most pronounced effects in the context of working memory electrophysiological investigations.

We hypothesized that chronic cannabis users (especially in the polydrug use context) would exhibit altered oscillatory dynamics in the alpha and theta range across brain regions known to be involved in the maintenance phase of the working memory (Sternberg) task and that this changed electrophysiology will be related to altered working memory functioning. We suspect that polydrug users will exhibit bigger differences in comparison to the control group than cannabis only users. Polydrug users’ brains are expected to exhibit more pronounced deviations from the standard brain due to a bigger chance of the potentially harmful substance to be consumed.

## Materials and Methods

### Participants

87 adults provided informed consent to take part in this study, with 84 included in behavioral analyses (reasons for exclusion of 3 participants are described below)—37 females and 47 male with mean age 29.44 ± 5.16 (details about participants are presented in Table 1) and 66 included in EEG data analyses (18 participants were excluded because of bad EEG signal). The research protocol was approved by the SWPS University Research Ethics Committee, and all participants gave written informed consent. We recruited thirty non-using controls (CG) who used cannabis on fewer than two occasions a year, and had not used in the preceding 90 days and 57 cannabis users, that were further divided in two subgroups which consist of 27 cannabis users (CU) using cannabis at least once a month (regular use) for at least 2 years (long-term use), and 30 cannabis polydrug users (PU) defined as using cannabis (at least once a month for at least 2 years) and using at least one other illicit drug in the last 3 months (detailed information about a kind of illicit drugs in supported in Supplementary Table 1). Our inclusion criteria were: 21–42 years of age; normal or corrected to normal vision; no history of brain injury, no diagnosis of neurological disease, no usage of psychotropic medications. Additional criteria for cannabis users were as follows: using cannabis at least once a month (regular use) and for at least 2 years (long-term use); negative results in screening test for cannabis use disorder [measured as ≤ 12 points at The Cannabis Use Disorder Identification Test–Revised (CUDIT-R; Adamson et al., 2010)]. It is important to note that we invited participants who declared cannabis use only (and no other illicit drugs) while recruiting to study, however, analyses of collected data in lab settings and hair sample analyses revealed polydrug use patterns in more than half of cannabis users. That is why we decided to include them in a study as a separate group which constitute a representative sample of cannabis users (Mitchell and Plunkett, 2000; Carlson et al., 2005; Lynskey et al., 2006; World Health Organization, 2016). Participants were screened for diagnosed psychiatric disorders based on self-declaration of the presence of a diagnosis by a mental health specialist, eight participants reported depression or anxiety (2 CG, 2 CU, and 5 PU), all other participants reported no psychiatric disorders. All the participants were right handed and spoke Polish as their primary language.

**TABLE 1:** Demographic and intelligence characteristics of the groups.

**Group (*n* = 84)**	**Controls (*n* = 27)**	**Cannabis users (*n* = 27)**	**Polydrug users (*n* = 30)**	**Three group comparisons**	
				**Statistic**	***P***
Female (%)	51.85	48.15	33.33	2.489**^a^**	0.325
Diagnosed psychiatric disorders (%)	7.41	7.41	16.64	1.728**^a^**	0.421
Age	28.67 ± 4.59	31 ± 6.17	28.73 ± 4.44	1.859**^b^**	0.162
Highest level of education (Years)	16.9 ± 1.85	17 ± 2.21	16.2 ± 1.96	1.155**^b^**	0.32
**Verbal intelligence quotient**					
WAIS scores Vocabulary	13.44 ± 1.93	13.11 ± 1.99	12.97 ± 1.75	0.473**^b^**	0.625
WAIS scores similarities	12.89 ± 1.97	13.11 ± 2.23	12.77 ± 1.85	0.212**^b^**	0.81
WAIS scores digit span	11.89 ± 3.07	13.11 ± 2.76	13.03 ± 3.06	1.454**^b^**	0.24
**Fluid intelligence quotient**					
WAIS scores matrix reasoning	12.93 ± 2.38	13.15 ± 2.41	13.07 ± 2.24	0.062**^b^**	0.94
WAIS score block design	12.78 ± 2.72	13.37 ± 2.65	13.4 ± 2.88	0.446**^b^**	0.642

*^a^χ2-test, degrees of freedom for each variable: sex df = (2); diagnosed psychiatric disorders df = (2).*

*^b^One-way ANOVAs df = (2.81).*

*There were no significant differences between cannabis users (CU), polydrug users (PU) and control group (CG) in any demographic characteristic nor IQ measurement.*

Cannabis users were included in the CU group if they reported regular and long-term cannabis use and, additionally, hair sample analysis detected no other drug metabolites [from analysis of hair samples reflecting past 3-month exposure: THC + (*n* = 12); no cannabinoid metabolites detected (*n* = 15)]. Cannabis polydrug users were assigned to the PU group, if they reported in self-assessment regular and long-term cannabis use and hair sample analysis reflected other drug metabolites [from analysis of hair samples reflecting past 3-month exposure: THC + (*n* = 20); 1 ≤ other illicit drug metabolites detected (*n* = 30)]. The most popular drugs used among PU group members were MDMA (*n* = 18), cocaine (*n* = 10) and amphetamine (*n* = 4). Non-drug using controls (CG) reported no drug use in self-assessment and had no drug metabolites detected in hair samples (Binkowska et al., 2021). Three participants from CG were excluded from all analysis, because of psychotropic medication detected in hair sample analysis (this was not delivered by self-report measurement). Hair samples were not collected from nine participants from the non-drug using control group (these participants refuse to lose a big amount of hair—diameter of a pencil—required for hair sample analyses, because of esthetic reasons). The detailed results of hair samples analyses are in the Supplementary Table 1. While four participants reported shorter than 12 h abstinence since last cannabis use (2 PU and 2 CU), it was highly possible that they used cannabis at night preceding experimental sessions. That is why we decided to include them in behavioral and EEG analyses (2 CU and 1 PU, one of them was excluded because of bad EEG signal). As shown in Table 1, groups were demographically comparable, with no significant differences in verbal and fluid intelligence. Fluid intelligence was assessed using Matrix Reasoning and Block Design from WAIS-R. Verbal intelligence was assessed using Vocabulary, Similarities and Digit Span subscales from WAIS-R.

Eighteen participants were excluded from EEG data analyses due to technical problems with signal recording, 7 in CG, 3 in CU and 9 in PU. Overall, 66 participants were included in EEG analyses: 10 females and 11 males in CG, 12 females and 12 males in CU and five females and 12 males in PU, groups did not differ significantly in sex proportions [X(2) = 3.752; *p* = 0.153].

Participants were recruited *via* advertisement and social media and received the description of their IQ test score and a sample of their brain’s electrical activity for their participation.

### Substance Use Assessment

Substance use was assessed by the self-reported drug history questionnaire, which included cannabis and other drugs use patterns (Table 2). In our study, we have measured the amount of cannabis consumed in joints, grams per week and puffs taken, however, we have missed other crucial factors that influence overall exposure, including THC:CBD ratio and cannabis potency (% THC). Moreover, illicit substance use over the last 3 months was examined by 3 cm-hair samples. The average concentration of each hair segment was calculated and used for the final analyses. Hair samples were analyzed for 512 drugs and their metabolites by an extremely sensitive and specific analytical technique—Liquid Chromatography Mass Spectrometry (LC-MS/MS). While there were missing answers for the question of cannabis use frequency in two participants in PU, hair sample analyses confirmed cannabis use.

**TABLE 2:** Substance use characteristics.

**Group (*n* = 84)**	**Controls (*n* = 27)**	**Cannabis users (*n* = 27)**	**Polydrug users (*n* = 30)**
**Alcohol, standard drinks per week,%**			
0	3.7	0	0
<1	44.4	48.2	43.3
1–3	37	48.2	33.3
4–6	14.8	3.7	20
7–14	0	0	3.3
14<	0	0	0
**Tobacco, %**			
No	74.1	44.4	56.7
Occasionally	25.9	40.7	26.7
Regullary	0	14.8	16.7
**Cannabis use pattern**			
Onset age, years mean	—	21.4 ± 4.68	19.7 ± 3.25
Duration, years mean (SD)	—	9.04 ± 7.09	8.28 ± 4.46
**Frequency of cannabis use (% of subjects) lifetime**			
0	81.5	3.7	6.7
Less than twice a year	18.5	0	3.3
2–3 times per month	0	0	6.7
1–3 times per week	0	29.6	23.3
3–6 times per week	0	37	23.3
Daily	0	29.6	36.7
No answer	0	0	0
**Frequency of cannabis use within past 30 days**			
0	100	7.4	10
2–3 times per month	0	7.4	3.33
1–3 times per week	0	29.6	23.3
3–6 times per week	0	33.3	33.3
Daily	0	18.5	30
Dose in puffs per one use mean (SD)		7.42 ± 3.05	7.38 ± 2.7
**Dose in grams per week (%)**			
Less than 1 g	0	38.5	28.6
1–2 grams	0	38.5	21.4
3–5 grams	0	23.1	39.3
>5 grams	0	0	10.7
**Time since last cannabis use (%)**			
<12 h	0	7.7	7.1
12–24 h	0	46.2	57.1
1–3 days	0	15.4	25
3–7 days	0	19.2	7.1
7–14 days	0	3.9	0
>14 days ago	0	7.7	3.6
**Other illicit drug use in last 30 days (% of subjects)**			
0	100	100	60
1 time per month	0	0	33.3
2 ≤ per month	0	0	6.7

*It is important to note that the ordinal data on substance use are presented in table in percentages, however, during analyses (while performing series of Mann–Whitney *U* or Kruskal–Wallis *H* tests) they were coded numerically.*

### Procedure

Researchers collecting data were blind to the group status and had no knowledge of the illicit substance use by the participants. Participants were asked to refrain from cannabis and other psychoactive substance use 12 h before attending the assessment session to ensure that examination would occur while they were not intoxicated. The abstinence was verified *via* the self-reported time and date of last use, and no observable signs of intoxication. Participants completed a short demographics questionnaire and answered questions about their drug use in a separate room to protect their privacy. Then, subjects performed a shortened version of Wechsler Adult Intelligence Scale—Revised including five subtests (Wechsler, 1981; Brzeziński and Hornowska, 1998). A 3 cm hair sample was collected from each participant (from the scalp).

Prior to the beginning of the experimental task, participants were verbally instructed as to what they would be experiencing and were shown what the procedure of EEG electrode mounting entails. Then participants were brought into a laboratory setting and seated in front of a 24 inch BenQ XL2411Z computer monitor (1,920 × 1,080 resolution, 100 Hz refresh rate) at a distance of 60 cm. Electrodes were then mounted and participants were briefly shown the EEG signal and explained how it is affected by eye blinks and muscular movements, which was a part of the procedure aimed at minimizing the amount of artifacts in the signal. The procedure was then started, and upon its completion subjects were provided with a place to wash their hair. The entire procedure lasted no more than 3 h.

#### Working Memory Task

**FIGURE 1:**
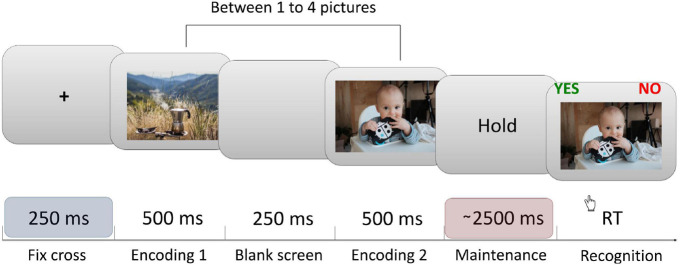
Sternberg task’s trial structure. Each trial began with a central fixation cross presented for 250 ms, followed by a sequential presentation of between one to four pictures (encoding phase). This was followed by the maintenance (delay) period lasting between 2,500 to 2,800 ms (randomly). Finally, after the delay, a probe image was shown. Participants were instructed to respond whether the probe was or was not presented during the encoding period that immediately preceded the probe. All EEG data analysis shown in this article were computed from the last ∼1.5 s (1,000–2,450 ms) interval of the maintenance period in order to avoid the activity evoked by the last image presentation. Each participant completed 120 trials of the task.

Participants performed a modified Sternberg task, with images as material for memorization—five pictures were randomly selected for each participant and were used as material during the procedure (Figure 1), for every trial a subset of pictures was randomly selected from the initial five pictures set. The participants had to memorize between 1 and 4 pictures, what resulted in four levels of memory load. 120 trials (30 per memory load) were presented, each trial presented between 1 and 4 pictures sequentially (encoding phase). Then, after 2.5–2.8 s (randomly) of maintenance period, subjects were presented with another picture and asked if they had seen it or not in the last presentation (recognition). Participants responded by pressing marked buttons on a keyboard. We asked participants to respond as fast as possible. Stimulus presentation and recording of responses were attained using PsychoPy (v1.85.6; Peirce, 2007). Performance accuracy was expressed as the percentage of correct answers. The average RTs were further broken down into a per-item search time and a constant. Per-item search time characterizes the time needed to compare a probe to the items in memory. For each individual and each condition, a regression function was fitted to the RT values as a function of memory loads (memory set sizes 1 to 4): y = ax + b, where a = per-item search time, x = memory load, and b = the constant (Sternberg, 1975; Jensen and Lisman, 1998).

#### EEG Recordings and Analysis

A 64-channel SynAmps RT Neuroscan EEG amplifier and BrainProducts actiCap Ag/AG-Cl active electrode set were used to record brain activity during task performance. All channels were recorded at 1,000 Hz sampling rate. Impedances were held below 15 kΩ. All data was preprocessed offline using the MATLAB environment and EEGlab (Delorme and Makeig, 2004) software package. Data were first high-pass filtered at 0.1 Hz, then bad channels were interpolated and signal was re-referenced to a common average. Data were segmented into epochs covering the time from 0.5 s before to 2.49 s after the onset of the fixation display (the maintenance period) of every trial. Incorrect trials (where participants gave incorrect answers) were very rare and excluded from analysis. Movement artifacts were manually removed from the data, after which an independent component analysis (ICA) was applied for an eyeblink artifact rejection. A 500 ms baseline correction was applied (from 500 ms before epoch).

We estimated the power spectrum for each electrode and each subject, separately for each memory load (1, 2, 3, or 4). Power spectra had a frequency resolution of 1 Hz and were computed from the last ∼1.5 s (1,000–2,450 ms) interval of the maintenance period in order to avoid the activity evoked by the last image presentation. Mean power was extracted for the theta (4–8 Hz), alpha (8–13 Hz) and beta (13–28 Hz) bands and areas of interest described below.

We computed absolute theta power for Fz (FMT) and absolute power in alpha, beta and theta frequency bands for two posterior clusters: right (P4, P6, PO4, PO8) and left (P3, P5, PO3, and PO7). Posterior clusters were defined according to the literature and corresponding topographical maps, see Figure 4D (Jensen et al., 2002; Böcker et al., 2010; Hu et al., 2019; Proskovec et al., 2019; Pavlov and Kotchoubey, 2020).

**FIGURE 2:**
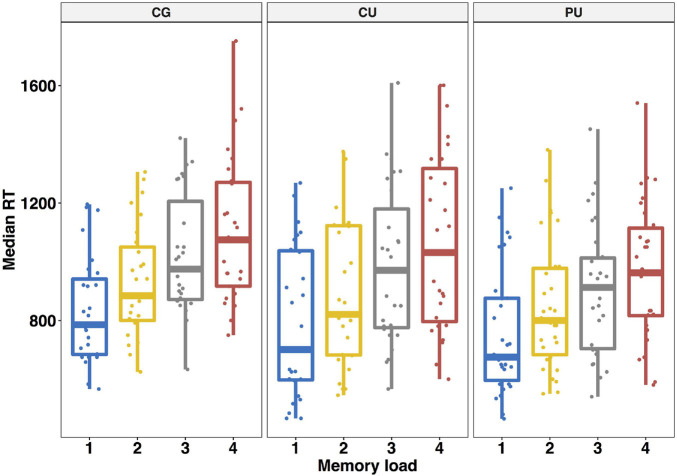
Working memory performance—averaged median reaction times (SD) in ms for each memory load in Sternberg task for control group (CG), cannabis users (CU) and polydrug users (PU).

**FIGURE 3:**
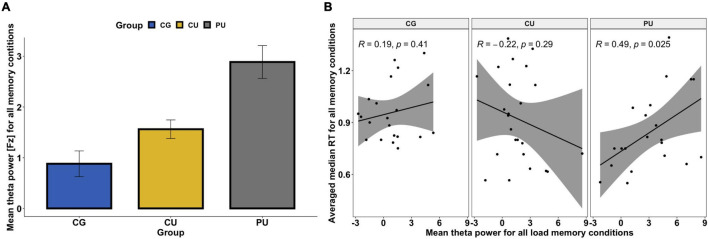
**(A)** Mean absolute theta (4–8 Hz) power for all memory loads for control group (CG), cannabis users (CU) and polydrug users (PU). There were significant differences between CG and PU (*p* < 0.05). No significant load effect was observed for the frontal midline (FM) theta power. **(B)** Association between mean theta power (Fz) averaged across all memory loads and averaged reaction time across all memory conditions (for correct answers only) for each group separately.

**FIGURE 4:**
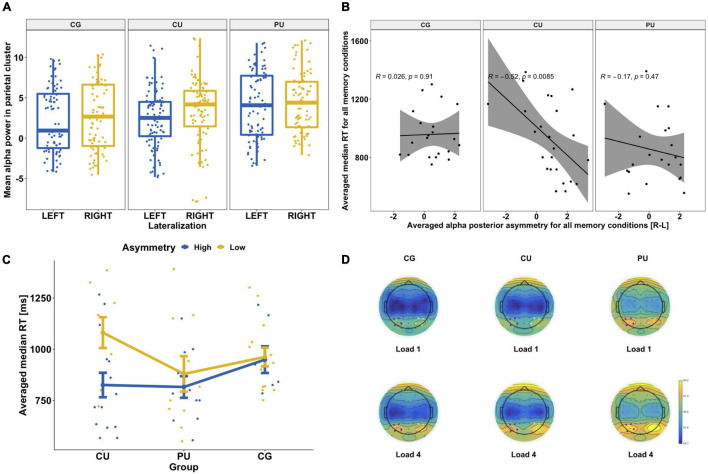
**(A)** Hemispheric asymmetries in posterior alpha power for each group separately: control group (CG), cannabis users (CU) and polydrug users (PU). There was significantly higher alpha power in the right posterior cluster [P4, P6, PO4, and PO8] than left [P3, P5, PO3, and PO7] for each group. **(B)** Association between posterior alpha asymmetry averaged across all memory loads and averaged reaction time across all memory conditions (for correct answers only) for each group separately. **(C)** Differences in RT depending on alpha power asymmetry level (high vs low) for three groups: CG, CU and PU. Both RT and alpha asymmetry power were averaged across all memory loads. **(D)** Topographic representation of alpha power (8–13 Hz) distribution during WM maintenance phase (1,000–2,450 ms) for two memory loads (1 and 4) for each group. Blue dots indicate the location of the right posterior cluster and purple dots indicate the location of the left posterior cluster.

### Statistical Analysis

Statistical analyses were performed using R software, version 4.0.2. Group comparisons for demographic, general functioning, intelligence quotient and substance use were conducted with a series of *t*-tests or ANOVAs for continuous variables, Mann–Whitney U or Kruskal–Wallis H tests for most substance use measures (as ordinal scales were used) and a χ2-test for categorical variables.

ANOVAs (one-way or repeated measures, as required) were used to analyze the behavioral and neurophysiological data including the between group variable (three levels: CG vs CU vs PU) and the within group variables: load (four levels: 1 vs 2 vs 3 vs 4) and hemisphere (two levels: left vs right) for posterior cluster analyses. For behavioral data the memory performance (RT and accuracy) was the outcome variable. For electrophysiological data the mean power for theta (4–8 Hz), alpha (8–13 Hz) and beta (13–28 Hz) frequency bands were the outcome variables.

Moreover, as load-dependent posterior alpha asymmetry (left < right) is a common finding in verbal and visual working memory studies (for review: Pavlov and Kotchoubey, 2020), we calculated asymmetry value for posterior alpha as follow: right posterior cluster—left posterior cluster (P4/3, P6/5, PO4/3, PO8/7) for mean alpha power in maintenance period (1,000–2,450 ms) for each memory load for each participant separately and included in analysis as dependent variable.

*Post hoc* pairwise *t*-tests were performed in case of significant main effects or interactions to check the simple effects, with Bonferroni correction for multiple comparisons. Correlations between frequency band power (alpha, theta or beta) during maintenance phase and task performance were performed using Pearson’s coefficient. In all tests, a *p*-value of less than 0.05 was considered statistically significant. To estimate effect sizes, we used η^2^. Data were presented as mean ± SD in text/tables and mean ± SEM in figures.

## Results

### Substance Use

Table 2 shows cannabis and other substance use self-report provided by participants.

Kruskal–Wallis H tests revealed no significant difference in tobacco H(2) = 4.142, *p* = 0.126) and alcohol use patterns H(4) = 3.362, *p* = 0.499, between CG, CU and PU. Comparison between CU and PU on cannabis use pattern shown no significant differences in cannabis use patterns: onset age *t*(49) = 1.483, *p* = 0.144, duration *t*(46) = 0.451, *p* = 0.654, dose in puffs per one use *t*(48) = 0.059, *p* = 0.953 (series of *t*-tests has shown), lifetime frequency of use *Z* = −0.250, *p* = 0.802, frequency of use in last 30 days *Z* = −0.555, *p* = 0.579, dose in grams per week *Z* = −1.764, *p* = 0.078 and time since last cannabis use *Z* = −1.052, *p* = 0.293 (series of Mann–Whitney *U* tests).

### Behavioral Results

#### Overall Sternberg Task Performance

Generally, participants performed very well on Sternberg task: 97.05% ± 2.9 (average performance accuracy for CU 97.2% ± 3.17, CG 96.88% ± 2.33 and PU 97.06% ± 3.18) with averaged median RT 911.37 ± 221 ms for correct trials only.

Consistent with previous reports of Sternberg task, accuracy decreased [*F*(3, 83) = 29.05, *p* < 0.0001, η^2^ = 0.126] and response time increased as a function of memory load [*F*(3, 83) = 131.5, *p* < 0.0001, η^2^ = 0.133]. Averaged median response times for whole participants were: 1-picture: 794 ms, 2-pictures: 887 ms, 3-pictures: 969 ms, 4-pictures: 1,041 ms. Averaged median RT for each load for each group are shown on Figure 2.

#### Between Group Comparisons—RT, Per-Item Search Time and a Constant

In order to investigate whether reaction times in Sternberg task vary between three groups we performed a 4 (memory load: 1 vs. 2 vs. 3 vs. 4) as a within-participant factor × 3 (substance use: CG vs. CU vs. PU) as a between-participant factor repeated—measures ANOVA on individuals’ median reaction time for each memory load. The main effect of memory load was significant [*F*(3,81) = 132.59, *p* < 0.0001, η^2^ = 0.133] unlike a main effect of group [*F*(2,81) = 1.61, *p* = 0.206, η^2^ = 0.03] or the interaction effect [*F*(6,81) = 1.342, *p* = 0.24, η^2^ = 0.003].

After breaking down the RTs into per—item search time and a constant, the negative values of delta RT were removed from this analysis as it imply decreasing RT with load in particular participant and were rare (5 participants: CG: *n* = 3; CU: *n* = 1; PU: *n* = 1). There were no significant differences in the per-item search time between three groups [*F*(2,76) = 2.612, *p* = 0.08, η^2^ = 0.064] nor for the constant [*F*(2,76) = 0.504, *p* = 0.606, η^2^ = 0.013].

Average per-item search time (ms) for each group was as follows CG 121 ± 54.5, CU 118 ± 56.7, PU 90.2 ± 51.7; and average constant (ms) CG 779 ± 230, CU 717 ± 266, PU 722 ± 236.

#### Between Group Comparisons—Accuracy

Next, in order to investigate whether accuracy in Sternberg task vary between three groups we performed a 4 (memory load: 1 vs. 2 vs. 3 vs. 4) as a within-participant factor × 3 (substance use: CG vs. CU vs. PU) as a between-participant factor repeated—measures ANOVA on individuals’ accuracy for each memory load. The main effect of memory load was significant [*F*(3,81) = 28.675, *p* < 0.0001, η^2^ = 0.125] unlike a main effect of group [*F*(2,81) = 0.091, *p* = 0.913, η^2^ = 0.001] or the interaction effect [*F*(6,81) = 0.467, *p* = 0.832, η^2^ = 0.004].

Therefore, group (and the substance use) did not determine participants’ speed of answering and accuracy.

### Frontal Midline Theta

First, repeated-measures ANOVA, with load (four levels: 1 vs. 2 vs. 3 vs. 4) as a within-participant factor, and group (three levels: CG vs. CU vs. PU) as a between-participant factor, was performed on the mean absolute theta power in Fz. ANOVA analysis revealed that there were neither a main effect of load [*F*(3, 63) = 1.327, *p* = 0.267, η^2^ = 0.001, nor load × group interaction [*F*(6, 63) = 0.629, *p* = 0.707, η^2^ = 0.001]. However, the significant main effect of the group was observed, *F*(2, 63) = 4.4221, *p* = 0.019, η^2^ = 0.113]. Bonferroni corrected *post hoc* tests revealed that there were significant differences only between CG and PU—the absolute FMT power was higher for each memory load (1, 2, 3, and 4) in PU than in CG (*p* < 0.05 for all). Figure 3A illustrates the mean FMT absolute power averaged across all loads for each group separately.

#### Correlations Between Memory Performance and Frontal Midline Theta

As FMT is associated with memory performance (Böcker et al., 2010; Brzezicka et al., 2019), Pearson’s correlation coefficients between performance metrics (RT and accuracy) and theta power at electrode Fz were calculated for all participants and in each group separately as significant differences between groups were observed in FMT power (Figure 3A and Table 3). While there were no significant relationships between FMT power and RT (*r* = 0.059, *p* = 0.64) for all participants, the significant positive correlation was found for the PU group (*r* = 0.49, *p* = 0.025)—the higher the FMT power, the slower the RT (see Figure 3B). Pearson correlation analyses revealed no significant association between FMT power and accuracy nor for all participants (*r* = −0.03, *p* = 0.807) nor for any of the groups separately (Table 3).

**TABLE 3:** Pearson coefficients for correlations (with *p* value reported) between RT and accuracy and frontal midline theta (FMT) during maintenance, at all WM loads and averaged across all memory loads for all participants and each group separately: control group (CG), cannabis users (CU), and polydrug users (PU).

	**All participants**		**CG**		**CU**		**PU**	
	**RT**	**Accuracy**	**RT**	**Accuracy**	**RT**	**Accuracy**	**RT**	**Accuracy**
FMT								
Load 1	0.07 *p* = 0.5768	0.0167 *p* = 0.8943	0.264 *p* = 0.2483	0.393 *p* = 0.078	−0.21 *p* = 0.3246	−0.064 *p* = 0.7657	0.409 *p* = 0.06579	0.009 *p* = 0.9695
Load 2	0.06 *p* = 0.6312	0.009 *p* = 0.9417	0.104 *p* = 0.6524	0.048 *p* = 0.835	−0.106 *p* = 0.6224	−0.36 *p* = 0.0843	0.361 *p* = 0.1076	0.235 *p* = 0.3048
Load 3	−0.0159 *p* = 0.8993	−0.074 *p* = 0.54	0.081 *p* = 0.7264	0.249 *p* = 0.2755	−0.247 *p* = 0.2438	−0.188 *p* = 0.38	**0.501** *p* = 0.0207	−0.135 *p* = 0.5604
Load 4	0.054 *p* = 0.6661	0.009 *p* = 0.9397	0.186 *p* = 0.4202	0.217 *p* = 0.3451	−0.261 *p* = 0.2179	0.127 *p* = 0.5555	**0.588** *p* = 0.0051	−0.387 *p* = 0.0827
Averaged for all loads	0.059 *p* = 0.6377	−0.031 *p* = 0.8074	0.189 *p* = 4121	0.25 *p* = 0.2747	−2.225 *p* = 0.2907	−0.163 *p* = 0.4463	**0.487** *p* = 0.0252	−0.061 *p* = 0.7943

*Coefficients are indicated in bold for *p* < 0.05; highlighted coefficient almost reaches *p* = 0.05.*

### Posterior Alpha Power

Repeated-measures ANOVA, with load (four levels: 1 vs. 2 vs. 3 vs. 4) and hemisphere lateralization (two levels: left vs right) as within-participant factors, and group (three levels: CG vs. CU vs. PU) as a between-participant factor, was performed on the mean absolute alpha power.

ANOVA analysis revealed that there were significant main effects of load [*F*(3, 63) = 14.398, *p* < 0.0001, η^2^ = 0.0047], lateralization [*F*(1, 63) = 55.745, *p* < 0.0001, η^2^ = 0.0061] and lateralization × group interaction [*F*(2, 63) = 3.149, *p* = 0.044, η^2^ = 0.0007]. There were no significant group effect [*F*(2, 63) = 1.187, *p* = 0.312, η^2^ = 0.0341] nor group × load [*F*(6, 63) = 0.478, *p* = 0.825, η^2^ = 0.9999], load × lateralization [*F*(3, 63) = 0.475, *p* = 0.7, η^2^ = 0.0002] or group × load × lateralization effect [*F*(6, 63) = 0.11, *p* = 0.1, η^2^ = 0.0001].

The load effect was observed where alpha power was increasing with loads. Bonferroni corrected *post hoc* tests revealed significant differences in alpha power in posterior clusters between loads: 1 and 2, 1 and 3, 1 and 4, 2 and 3, 2 and 4 for all participants (*p* < 0.05).

*Post hoc* tests revealed that there were significant differences between right and left posterior alpha power in each group (*p* < 0.05 for all), see Figure 4A.

#### Correlations Between Memory Performance and Alpha Asymmetry Indicator

Based on observed posterior alpha lateralization, we calculated the alpha asymmetry indicator (see in statistical analyses section) to investigate if it corresponds to behavioral measures (Table 4). Pearson correlation analyses revealed significant association between alpha asymmetry during the maintenance period and RT averaged across all WM loads for all participants (*r* = −0.268, *p* = 0.03), but not for accuracy (*r* = 0.094, *p* = 0.455). Figure 4B shows correlations between posterior alpha asymmetry and averaged RT for each group separately, there is significant correlation only for CU (*r* = −0.52, *p* = 0.009).

**TABLE 4:** Pearson coefficients for correlations (with *p* value reported) between RT and accuracy and alpha posterior asymmetry (P4/3, P6/5, PO4/3, and PO8/7) during maintenance, at all WM loads and averaged across all memory loads for all participants and each group separately: control group (CG), cannabis users (CU), polydrug users (PU).

	**All participants**		**CG**		**CU**		**PU**	
	**RT**	**Accuracy**	**RT**	**Accuracy**	**RT**	**Accuracy**	**RT**	**Accuracy**
**Alpha asymmetry**								
Load 1	−0.189 *p* = 0.1279	0.142 *p* = 0.2562	0.233 *p* = 0.3084	−0.048 *p* = 0.8353	−**0.43** *p* = 0.0347	0.135 *p* = 0.5299	−0.212 *p* = 0.3561	0.305 *p* = 0.1786
Load 2	−0.231 *p* = 0.0626	0.0005 *p* = 0.997	0.101 *p* = 0.6625	−0.031 *p* = 0.8954	−**0.44** *p* = 0.0318	−0.038 *p* = 0.8602	−0.196 *p* = 0.3958	0.021 *p* = 0.9268
Load 3	−**0.279** *p* = 0.0236	0.048 *p* = 0.7031	−0.183 *p* = 0.4274	−0.032 *p* = 0.8906	−**0.55** *p* = 0.005	0.171 *p* = 0.4232	−0.032 *p* = 0.8895	−0.08 *p* = 0.7303
Load 4	−0.197 *p* = 0.1128	0.116 *p* = 0.3543	−0.15 *p* = 0.515	−0.046 *p* = 0.8414	0.39 *p* = 0.058	0.226 *p* = 0.2881	0.055 *p* = 0.8132	0.076 *p* = 0.7439
Averaged for all loads	−**0.268** *p* = 0.0297	0.094 *p* = 0.4547	0.026 *p* = 9105	−0.071 *p* = 0.7613	−**0.524** *p* = 0.0085	0.231 *p* = 0.2764	−0.168 *p* = 0.4677	−0.021 *p* = 0.9278

*Coefficients are indicated in bold for *p* < 0.05; highlighted coefficient almost reaches *p* = 0.05.*

#### Differences in RT Depending on Alpha Power Asymmetry Level

To further examine posterior alpha asymmetry effect and its association with RT, we divided subjects to high or low asymmetry groups according to median split calculated based on all participants’ asymmetry results (median = 0.79). Figure 4C shows the differences between groups with low and high alpha posterior asymmetry level in each studied group for averaged RT. Series of U Mann–Whitney tests revealed that there were significant differences between asymmetry level and averaged median time only in CU (Z = 27, *p* = 0.015) with mean RT: 825 ± 231 ms for high asymmetry and 1,081 ± 225 ms for low asymmetry.

## Discussion

This study extends upon previous research on the effects of chronic cannabis use on working memory and demonstrates that the neural processes associated with WM are altered in regular cannabis users even when they do not display behavioral evidence of WM impairment. Our results clearly indicate that it is important to make a distinction between regular cannabis users and polydrug regular cannabis users as those groups are characterized by different oscillatory patterns in comparison to non-using controls. We have observed the highest posterior alpha asymmetry in the CU group, compared to PU and CG groups. At the same time the PU group has shown the highest FMT power compared to CU and PU groups. These two physiological indicators were correlated with WM performance (RT), but the correlation was group specific—posterior alpha asymmetry was negatively related to RT in the CU group, while FMT power was positively related to RT in the PU group. There were no differences in performance level between groups. The posterior asymmetry effect was frequency-specific and visible exclusively in the alpha band, we did not identify associations with behavioral indicators in theta or beta range (see additional statistical analyses in Supplementary Material).

### Behavioral Performance

In line with previous working memory studies (e.g., Kanayama et al., 2004; Jager et al., 2006; Nestor et al., 2008; Smith et al., 2010; Cousijn et al., 2014; Ma et al., 2018), no group difference was found in the behavioral performance during the WM task between cannabis users and non-using controls. However, in other studies chronic cannabis use has been linked to working memory impairments (e.g., Gruber et al., 2012; Becker et al., 2014; Thames et al., 2014).

There may be several important reasons for inconsistent results across studies—the range of tasks used to assess cognitive functioning (and probe neural processes underlying them), different cannabis use patterns (e.g., age of onset, lifetime exposition, frequency) further complicated by difficulty in standardizing these patterns, varied inclusion and exclusion criteria for other substance use.

At the same time, findings from WM studies highlight the importance of employing neuroimaging and electrophysiological techniques to complement classic neuropsychological assessments, as changes in neurophysiological activity seems to be frequent among cannabis users even when behavioral performance is intact (Sagar and Gruber, 2019). We cannot exclude that the altered brain activity could be detectable before cognitive impairment comes up in chronic drug users.

### Posterior Alpha Asymmetry

Significant alpha asymmetry with higher activity in the right than in the left posterior cluster during the maintenance phase was observed in all groups. It is a common effect observed in previous studies (for review: Pavlov and Kotchoubey, 2020). The possible interpretation of this finding is that maintenance of information presented visually and easy to verbalize (as used in this study) involves two simultaneous processes that support successful information maintenance. Suppression of visual input by disengaging visual cortex and activation of the language cortex for verbal rehearsal. Inhibition of distracting visual stimuli increases alpha power/activity in both hemispheres. While, at the same time verbalization as a memory strategy involves left posterior regions, which is manifested by suppression of alpha power in the left hemisphere (Pavlov and Kotchoubey, 2020).

The posterior alpha asymmetry was associated with RT only in the CU group, where higher alpha asymmetry corresponded to faster RT. The possible explanation of observed relationship between posterior alpha asymmetry and RT in the CU group is that higher asymmetry would reflect more effective information maintenance process (higher alpha power in right hemisphere = more efficient sensory gating mechanism and lower alpha power in left hemisphere = enhanced verbal rehearsal). As shown in Figure 4C there was a similar (non-significant) tendency in the PU group, and no such tendency in the CG group.

Previous fMRI study conducted by Jager et al. (2006) has shown that cannabis users displayed an abnormality in the left superior parietal cortex (increased activity, which is related to lower alpha power), despite equivalent performance to controls on a working memory task. The left superior parietal cortex plays a role in short-term storage and retrieval of verbally coded material (Smith and Jonides, 1997; Jansma et al., 2001), so it could partially support our interpretation as the results we obtained are similar.

Another explanation of significant association between posterior alpha asymmetry and RT only in the CU group may be due to specific compensatory task strategy involving higher level of verbalization. The verbal rehearsal could be less frequent in PU, and CG engaged it at even lower level. There were no significant differences in RT (or accuracy) between groups, so all strategies seem to be equally effective. However, we did not ask participants after completing the WM task about the memory strategy they used and it is worthy to consider in future studies.

The parahippocampal dysfunction during WM encoding and retrieval has been also observed in fMRI studies in cannabis users (Nestor et al., 2008; Becker et al., 2010a), suggesting the association between a higher frequency of cannabis use and increased activity in the left parahippocampal gyrus (which might reflect functional compensation to maintain cognitive functioning) (Becker et al., 2010a). While we did not observe correlation between frequency of cannabis use and psychophysiological indicators it is possible that this measurement was inaccurate in our study and we cannot completely exclude the differences between CU and PU, that could impact obtained results.

Moreover, some studies using modified Sternberg task showed alpha power increases in posterior (visual) brain regions with WM load during retention, this effect was observed in our study as well (Jensen et al., 2002; Meltzer et al., 2008; Proskovec et al., 2019).

As mentioned in the introduction, it is assumed that alpha activity is a manifestation of a sensory input suppression from the visual stream to prevent disruption to WM maintenance occurring in frontal brain areas (Jensen and Mazaheri, 2010). Studies using lateralized WM tasks support the view on posterior alpha asymmetry’s role in sensory inhibition. The alpha power reduction in the contralateral compared to the ipsilateral occipital cortex is in line with notions that consider hemispheric alpha power asymmetries as a consequence of visual input inhibition (Figueira et al., 2020).

### Frontal Midline Theta

Significant differences in frontal midline theta (FMT) power were observed between polydrug users and control group (but not in the CU group) but again we saw no differences in memory performance. PU has shown significantly higher FMT power for each memory load than CG.

Findings from previous research in cannabis users are mostly consistent showing the association between working memory impairments associated with hyperactivation and hyperconnectivity of working memory circuits particularly in the prefrontal cortex (Kanayama et al., 2004; Becker et al., 2010b; Colizzi et al., 2015; Tervo-Clemmens et al., 2018; Bloomfield et al., 2019). The important factors that impact these effects were total cannabis exposure (Tervo-Clemmens et al., 2018), that may be further mediated by CB1 receptor genotype (Colizzi et al., 2015). Moreover, Tervo-Clemmens et al. (2018) has shown that total cannabis use is positively associated with greater prefrontal activation during WM maintenance and shows a trend toward lower WM performance. This is in line with previous studies suggesting compensatory-like prefrontal activation in groups with poorer executive function performance, including cannabis uses (Ordaz et al., 2013; Taurisano et al., 2016; Van Snellenberg et al., 2016). We cannot conclude that the observed neurocognitive deficits exist before substance/cannabis use, further prospective longitudinal studies are needed to investigate this hypothesis.

The most popular other illicit psychoactive substance among PU in our study was MDMA. MDMA is the most commonly used drug in polydrug context with cannabis (Daumann et al., 2004; Scholey et al., 2004; European Monitoring Centre for Drugs and Drug Addiction, 2018).

Previous research has shown a similar effect of increased FMT power during memory tasks in MDMA and cannabis users (Buchert et al., 2001; Simon and Mattick, 2002; Herning et al., 2005). The authors suggest that higher FMT activity may indicate an imbalance in the excitation-inhibition homeostasis in the cortex or a deficiency in the information-processing capacity of the central nervous system (Buchert et al., 2001; Simon and Mattick, 2002; Herning et al., 2005). MDMA acts *via* serotonergic receptors and may cause disturbances of serotonergic pathways (Benningfield and Cowan, 2013). The high density of 5-HT receptor in the prefrontal cortex, which is involved in WM processes may alter brain function in this area during WM tasks (Jager et al., 2008; Roberts et al., 2018). The other psychoactive substances used among PU, such as cocaine, amphetamine and others may interact in a complex way and cumulative effect may impact the further increased brain activity (Zilverstand et al., 2018; Khajehpour et al., 2019). Unfortunately, we did not collect detailed information about the nature of polydrug use, e.g., age of onset, lifetime use, amount and frequency of use that could have significant impact on results.

Moreover, a study by Kim et al. (2011) applying graph theory to diffusion tensor imaging and tractography has shown that cannabis users have less efficiently integrated global structural brain networks with altered regional altered local connectivity in the cingulate cortical regions. Cingulate cortex is one of the key regions engaged in executive function and working memory. It is possible that integrative processing among brain regions required for efficient and effective cognitive functioning is disrupted by cannabis use (however, it cannot be excluded that study participants were polydrug users). As the allocation of cognitive resources has often been associated with neural oscillations in the theta frequency range in medial prefrontal cortex (PFC) and anterior cingulate cortex (Mitchell et al., 2008; Cavanagh and Frank, 2014) known as FM theta activity, it can be manifested in observed altered FMT power in PU.

Additionally, previous EEG and fMRI studies have shown increased cortical activation during resting state condition and task performance in users of several drugs, also in a polydrug use context (Jager et al., 2008; Adamaszek et al., 2010; Roberts and Garavan, 2010; Roberts et al., 2018). This increased resting state cortical activation was observed in cannabis users as well, however, the objective drug use measurement (urine samples) was intended only for proving cannabis use, so the other psychoactive substances were not checked (Struve et al., 1998, 1999; Prashad et al., 2018). That is why, we can not exclude the possible impact of polydrug use on observed altered resting state brain activity in regular cannabis users.

As the FMT power during WM maintenance is considered as a neural marker of sustained and internally-focused attention (Raghavachari et al., 2001; Jensen and Tesche, 2002), the significantly higher FMT power in PU could indicate higher cognitive effort and attention engagement in completing the task, as there were no differences in task performance. What is more, the significant association between FMT power and RT was observed in PU, where RT was increasing linearly with FMT power during WM maintenance (Figure 3B). While these results may seem surprising, it is possible that increased cortical activation in PU and higher allocation of neural resources toward task-relevant neural processes exhibited by higher FMT power may mirror a loss of cortical efficiency and subjectively perceived higher level of task difficulty compared to non-using controls—which effect in slowing RT. Previous studies have shown similar positive correlation between theta power and reaction times in visual tasks in ADHD patients compared to healthy controls (Roh et al., 2015). The possible explanation is also higher engagement of cognitive control in both cases. The relationship between cognitive control and FM theta is supported by human and animal (primats) studies showing that activity in theta band in medial PFC increases with cognitive control demands (Tsujimoto et al., 2006, 2010; Cavanagh et al., 2011).

Moreover, we have not found FMT increase during maintenance phase with memory load in any group, similar results were observed in previous studies (Heinrichs-Graham and Wilson, 2015; Honkanen et al., 2015; Vandenbroucke et al., 2015; Ellmore et al., 2017). In the review article Pavlov and Kotchoubey (2020) suggest that FMT enhanced with memory load in verbal tasks, while this relationship is less common in visual WM tasks and our modified Sternberg task based on visual stimuli. The authors explain this discrepancies between stimuli modality by the temporal order hypothesis, which claims that building a temporal structure for the maintenance of multiple items in verbal WM is the key role of FMT.

### Strengths and Limitations

Several limitations of the current study should be noted. First, in research on the effects of chronic cannabis use on cognition and brain function the dosage is an important factor, however, almost impossible to evaluate correctly (especially in recreational cannabis users and in countries where such use is illegal). Unlike alcohol, there is no standardized measure of cannabis. Assessing exposure to cannabis and even comparing study results are further complicated by rising levels of cannabis potency, the mean THC concentration has increased dramatically over the last 10 years, from 8.9% in 2008 to 17.1% in 2017 (Chandra et al., 2019). That is why in our study we cannot assume that the lifetime amount of cannabis consumed had an effect on our results.

Secondly, the hair samples were not collected from nine participants from the control group. As the observed tendency among participants was rather to under-report drug use in cannabis users (mainly in case of other illicit drug use) we assumed their self-reports were reliable. However, the lack of self-declarative information about SSRI medication in some participants in CG left some uncertainty. It is important to note, that the hair sample analyses did not prove THC presence in all cannabis users, but it allowed to disqualify other illicit drug use in the CU group. Previous body of research showed that the sensitivity of THC detection in hair is almost 80% in heavy cannabis smokers compared to light and non- cannabis users, but fell to 55% in any cannabis users compared to non-cannabis users (Taylor et al., 2017). The big advantage of hair sample analysis is that it provides the opportunity to detect many drugs metabolites among much longer time-frames (3 months in our study), which makes it a suitable tool for long-term drug use assessment and can also be considered a strength of this study.

Third, there is always a risk of cannabis use prior to the study visit and consequently investigating acute instead of residual effects. We ask participants to refrain from cannabis and other psychoactive substance use 12 h before attending the assessment session to ensure that examination would occur while they were not intoxicated. While four participants reported last cannabis use shorter than 12 h, it is highly possible that they used cannabis at night preceding experimental sessions, as their measurements were in the morning and no participant has demonstrated any observable signs of intoxication. Fourth, the age of cannabis use onset in our participants could be considered as a limitation, because usually in cannabis research the age of onset is younger (during adolescence). That is why, our results may be not possible to generalize on cannabis users that started regular cannabis use much earlier. At the same time, our participants were middle-aged and have shown long lifetime cannabis use (∼10 years), giving us the opportunity to investigate higher cumulative effects of cannabis use. Fifth, the control group enrolled in our research consists of individuals with no cannabis or/and other drug use history and individuals with minimal use in their lifetime (<50 occasions). Such level is considered as acceptable, because it attenuates a potential cumulative effect of cannabis use (Sagar and Gruber, 2019). Sixth, we acknowledge that the group sizes were modest in our study, however, most neuroimaging investigations in cannabis research have similar sample sizes (Sagar and Gruber, 2019) and these samples appear to be large enough to detect between-group differences in neural oscillatory activity.

Although research (including our study) has clearly shown that chronic recreational use influences, rather slightly, brain function, future studies should emphasize the exploration of moderating factors as frequency and magnitude of cannabis use, potency of cannabis, lifetime use, and detailed pattern of other substance use. These factors are of great importance to fully understand the impact of cannabis on human neurocognitive functioning.

In summary, these findings extend upon previous research and demonstrate that the neural processes associated with WM processing are altered in regular cannabis users, differently depending on other drug use (polydrug) context. Regular cannabis users and polydrug cannabis users exhibit diverse neural oscillatory alterations, despite no impairment on the WM task.

## Data Availability Statement

The raw data supporting the conclusion of this article will be made available by the authors, without undue reservation.

## Ethics Statement

The studies involving human participants were reviewed and approved by the SWPS University Research Ethics Committee. The patients/participants provided their written informed consent to participate in this study. Written informed consent was obtained from the minor(s)’ legal guardian/next of kin for the publication of any potentially identifiable images or data included in this article.

## Author Contributions

AAB and AB: study design. AAB and NJ: data processing and data analysis. NG and AP-C: data analysis. AAB, AB, NJ, KK, NG, and AP-C: manuscript writing. All authors contributed to the article and approved the submitted version.

## Conflict of Interest

The authors declare that the research was conducted in the absence of any commercial or financial relationships that could be construed as a potential conflict of interest.

## Publisher’s Note

All claims expressed in this article are solely those of the authors and do not necessarily represent those of their affiliated organizations, or those of the publisher, the editors and the reviewers. Any product that may be evaluated in this article, or claim that may be made by its manufacturer, is not guaranteed or endorsed by the publisher.
