# Supplementary material for: Theta and Alpha Oscillatory Activity During Working Memory Maintenance in Long-Term Cannabis Users: The Importance of the Polydrug Use Context
Source: Front Hum Neurosci. 2021 Oct 18;15:740277. doi: 10.3389/fnhum.2021.740277 (PMC8558244; doi:10.3389/fnhum.2021.740277)
Supplement: Supplementary file 1 [file Data_Sheet_1.PDF]

Supplementary Material

Supplementary table 1. Substance use data - hair samples analyses results.

**Hair sample pos (number)**	**Control group n = 30**	**Cannabis users n = 27**	**Polydrug users n = 30**
THC	-	12	20
MDMA	-	-	18
LSD	-	-	1
Amphetamine	-	-	4
Methcathinone	-	-	2
Cocaine	-	-	10
Cathine	-	-	1
Sertraline	1	-	-
Paroxetine	1	-	-
Carbamazepine	1	-	-

Supplementary statistical analyses

Posterior beta and theta power

Additional comparisons of oscillatory activity within adjacent frequency ranges were conducted to check if the observed effect is specific for the alpha frequency band, including the theta (4–8 Hz) and beta range (13–28 Hz).
As before, repeated-measures ANOVA, with load (four levels: 1 vs. 2 vs. 3 vs. 4) and hemisphere lateralization (two levels: left vs right) as within-participant factors, and group (three levels: CG vs. CU vs. PU) as a between-participant factor, was performed on the mean absolute theta and beta power.

Posterior theta power

ANOVA analysis revealed that there were significant main effects of load [$F(3, 63) = 6.549, p < 0.0001, \eta^2 = 0.002$] and lateralization [$F(1, 63) = 27.592, p < 0.0001, \eta^2 = 0.0028$] on theta power. There were no significant group effect [$F(2, 63) = 2.693, p = 0.076, \eta^2 = 0.0748$] nor lateralization * group interaction [$F(2, 63) = 0.751, p = 0.473, \eta^2 = 0.0007$], group * load [$F(6, 63) = 0.308, p = 0.933, \eta^2 = 0.0002$], load * lateralization [$F(3, 63) = 0.653, p = 0.582, \eta^2 = 0.0002$] or group * load * lateralization effect [$F(6, 63) = 0.48, p = 0.823, \eta^2 = 0.0003$].

While further exploring lateralization effect, post-hoc tests revealed significantly higher theta power in the right than left posterior cluster for all participants ($p<0.0001$).

While further exploring load effect, Bonferroni corrected post-hoc tests revealed significant differences in theta power in posterior clusters between loads: 1 and 2, 1 and 3, 1 and 4 for all participants (p<0.001), with lower theta power for lower loads.

Posterior beta power

For posterior beta, ANOVA analysis revealed significant main effect of load [$F(3, 63) = 8.281, p < 0.0001, \eta^2 = 0.002$], almost significant lateralization effect [$F(1, 63) = 3.741, p = 0.0537, \eta^2 = 0.0003$] and significant lateralization * group interaction [$F(2, 63) = 10.219, p < 0.0001, \eta^2 = 0.0016$]. There were no significant main group effect [$F(2, 63) = 1.2, p = 0.308, \eta^2 = 0.0353$] nor group * load [$F(6, 63) = 0.122, p = 0.994, \eta^2 = 0.0001$], load * lateralization [$F(3, 63) = 0.879, p = 0.452, \eta^2 = 0.0002$] or group * load * lateralization effect [$F(6, 63) = 0.109, p = 0.995, \eta^2 = 0.0001$].

While further exploring lateralization * group interaction effect, Bonferroni corrected post-hoc tests revealed that there were significant differences between right and left posterior beta power only in CU (p<0.0001), with higher beta power in the right cluster.

While further exploring load effect, Bonferroni corrected post-hoc tests revealed significant differences in beta power in posterior clusters between loads: 1 and 2, 1 and 3, 1 and 4, 2 and 3, 2 and 4 for all participants (p<0.05), with lower beta power for lower load.

Correlations between memory performance and posterior theta and beta asymmetry

Similar to alpha asymmetry, the posterior asymmetry indicator was calculated for theta and beta band to investigate its association with memory performance. Pearson correlation analyses revealed no significant association nor between theta or beta asymmetry during the maintenance period and performance metrics (RT and accuracy) for all participants and in each group separately (supplementary table 2).

Supplementary table 2. Pearson coefficients for correlations (with p value reported) between RT and accuracy and theta and beta posterior asymmetry (P4/3, P6/5, PO4/3, PO8/7) during maintenance, at all WM loads and averaged across all memory loads for all participants and each group separately: control group (CG), cannabis users (CU), polydrug users (PU).

	**All participants**		**CG**		**CU**		**PU**	
	**RT**	**accuracy**	**RT**	**accuracy**	**RT**	**accuracy**	**RT**	**accuracy**
**Theta asymmetry**								
Load 1	-0.135<br>p=0.2803	**0.352**<br>p=0.004	-0.171<br>p=0.4579	0.17<br>p=0.4614	**-0.41**<br>p=0.045	0.34<br>p=0.1042	0.237<br>p=0.3013	**0.609**<br>p=0.003
Load 2	-0.147<br>p=0.2404	0.219<br>p=0.0774	0.213<br>p=0.3539	0.151<br>p=0.5132	**-0.47**<br>p=0.019	0.181<br>p=0.3986	0.161<br>p=0.4843	0.312<br>p=0.1684
Load 3	-0.037<br>p=0.7578	0.086<br>p=0.4918	-0.235<br>p=0.3061	0.309<br>p=0.1731	-0.29<br>p=0.172	0.021<br>p=0.9224	0.41<br>p=0.0669	0.048<br>p=0.8357
Load 4	-0.058<br>p=0.651	0.038<br>p=0.7601	-0.081<br>p=0.7261	0.163<br>p=0.4806	-0.24<br>p=0.255	0.119<br>p=0.5785	0.192<br>p=0.4035	-0.252<br>p=0.27

Averaged for all loads	-0.111 p=0.3735	0.171 p=0.1697	-0.088 p=0.7039	0.154 p=0.5048	-0.386 <u>p=0.062</u>	0.191 p=0.3718	0.259 p=0.2574	0.155 p=0.5023
**Beta asymmetry**								
Load 1	0.015 p=0.9074	0.062 p=0.6187	-0.006 p=0.9804	-0.057 p=0.8072	-0.314 p= 0.1346	0.132 p=0.5373	0.369 p=0.1	0.09 p=0.7038
Load 2	-0.073 p=0.5589	-0.04 p=0.7486	-0.129 p=0.5776	0.017 p=0.9402	-0.212 p=0.3197	-0.184 p=0.3881	0.118 p=0.611	-0.031 p=0.8948
Load 3	-0.006 p=0.961	-0.09 p=0.4746	-0.13 p=0.5737	0.141 p=0.5417	-0.22 p=0.3024	-0.215 p=0.3133	0.337 p=0.1353	-0.244 p=0.2858
Load 4	-0.094 p=0.4532	0.041 p=0.7446	-0.169 p=0.4636	-0.13 p=0.5729	-0.34 p=0.104	0.236 p=0.2674	0.241 p=0.2935	-0.095 p=0.682
Averaged for all loads	-0.049 p=0.6987	0.027 p=0.832	-0.106 p=0.6489	0.071 p=0.7605	-0.3 p=0.1547	0.083 p=0.7004	0.27 p=0.2376	-0.159 p=0.4909
